# Myogenesis controlled by a long non-coding RNA 1700113A16RIK and post-transcriptional regulation

**DOI:** 10.1186/s13619-022-00114-x

**Published:** 2022-04-03

**Authors:** Xin Fu, Sheng Li, Minzhi Jia, Bo Xu, Lele Yang, Ruimiao Ma, Hong Cheng, Wenjun Yang, Ping Hu

**Affiliations:** 1 Spine Center, Department of Pediatric Orthopedics, Xin Hua Hospital Affiliated to Shanghai Jiao Tong University, School of Medicine, Shanghai, 200092 China; 2 State Key Laboratory of Cell Biology, Shanghai Institute of Biochemistry and Cell Biology, Center for Excellence in Molecular Cell Science, Chinese Academy of Sciences, Shanghai, 200031 China; 3 Guangzhou Laboratory, Guangzhou, 510700 Guangdong China; 4 State Key Laboratory of Molecular Biology, Shanghai Institute of Biochemistry and Cell Biology, Center for Excellence in Molecular Cell Science, Chinese Academy of Sciences, Shanghai, 200031 China; 5 Institute for Stem Cell and Regeneration, Chinese Academy of Sciences, Beijing, 100101 China

**Keywords:** Long non-coding RNA (lncRNA), 1700113A16RIK, Muscle stem cell (MuSC) differentiation, Myocyte-specific enhancer binding factor 2 (MEF2D)

## Abstract

Long non-coding (lnc) RNA plays important roles in many cellular processes. The function of the vast majority of lncRNAs remains unknown. Here we identified that lncRNA-1700113A16RIK existed in skeletal muscle stem cells (MuSCs) and was significantly elevated during MuSC differentiation. Knockdown of 1700113A16RIK inhibits the differentiation of muscle stem cells. In contrast, overexpression of 1700113A16RIK promotes the differentiation of muscle stem cells. Further study shows the muscle specific transcription factor Myogenin (MyoG) positively regulates the expression of 1700113A16RIK by binding to the promoter region of 1700113A16RIK. Mechanistically, 1700113A16RIK may regulate the expression of myogenic genes by directly binding to 3’UTR of an important myogenic transcription factor MEF2D, which in turn promotes the translation of MEF2D. Taken together, our results defined 1700113A16RIK as a positive regulator of MuSC differentiation and elucidated a mechanism as to how 1700113A16RIK regulated MuSC differentiation.

## Background

Muscle regeneration is a highly coordinated process that includes sequential steps of activation of transcription factors named myogenic regulatory factors (MRFs) including MyoD, Myf5 and myogenin (MyoG) and other transcription factors such as Pax7 and Pax3 have been shown to play important roles in orchestrating the muscle regeneration processs (Asfour et al. 2018; Zammit 2017; Hernandez-Hernandez et al. 2017; Schmidt et al. 2019; Robinson and Dilworth 2018; Pourquie et al. 2018; Buckingham and Relaix 2015). Transcription regulation is critical for muscle regeneration and myogenesis. Besides transcriptional regulation mediated by transcription factors, recent studies have shown that DNA modification enzymes, RNA binding proteins, microRNA, and long non-coding RNA (lncRNA) are also essential for skeletal muscle regeneration (Yang et al. 2022; Wang et al. 2021; Fu et al. 2015; Yang and Hu 2018a, b). Understanding the regulatory network of skeletal myogenesis will contribute to the treatment of human muscle related diseases (Almada and Wagers 2016; Wang et al. 2019; Liu and Bassel-Duby 2015). LncRNAs serve as regulators in the proliferation of MuSCs, differentiation of myoblasts, and myoblast fusion to form multinucleated myotubes (Berkes and Tapscott 2005; Wang et al. 2018; Ballarino et al. 2016; Zhao et al. 2019). LncRNAs represent a class of transcribed RNA molecules with a length of more than 200 nucleotides and have no coding ability (Ghildiyal and Zamore 2009; Derrien et al. 2011). Recent studies have reported that lncRNAs regulate myogenesis through multiple mechanisms such as chromatin structure modification, transcription regulation, microRNA antagonizing, and translational regulation (Watts et al. 2013; Borensztein et al. 2013; Dey et al. 2014; Wang et al. 2013; Legnini et al. 2014). However, the identified lncRNAs in myogenesis are merely the tip of the iceberg, and a large number of lncRNAs in myogenesis remain to be explored.

The myocyte-specific enhancer binding factor 2 (Mef2) family proteins are critical transcription factors in muscle cells. Four members of the family have been identified in vertebrate, namely Mef2a, −2b, −2c, and -2d (Molkentin et al. 1995; Sebastian et al. 2013). They display distinct but overlapping temporal and spatial expression patterns in the embryo and adult tissue. The four Mef2 proteins share the conserved N-terminal MADS-box domain and MEF2 domain, but are divergent in the C-terminal transcriptional activation domains (Black and Olson 1998). Mef2a, −2c, and 2d have all been detected in somatic muscle during embryonic development (Edmondson et al. 1994). Mef2a, −2c, and -2d are required for myogenesis. In Mef2 knockout mice, the differentiation of muscle cells was found to be inhibited (Lilly et al. 1995; Bour et al. 1995; Liu et al. 2014).MyoD interacts with Mef2a, −2c, or -2d to synergistically activate differentiation related genes (Molkentin et al. 1995). As a member of Mef2 family, Mef2d has been reported that plays a key role in myogenesis (Sebastian et al. 2013; Liu et al. 2014; Runfola et al. 2015). Mef2d interacts with members of myogenic regulatory factors to activate expression of various myogenic related genes such as myogenin and miR206 (Penn et al. 2004; Sui et al. 2019).

Here, we identified a novel lncRNA, 1700113A16Rik, regulating muscle regeneration. 17000A16Rik was enriched in the cytoplasm of differentiated MuSCs and required for the differentiation of MuSCs. 1700113A16RIK paired with the complementary sequence of the 3’UTR region of Mef2d to facilitate its translation and therefore promotes MuSC differentiation.

## Results

### 1700113A16RIK expression is upregulated during MuSC differentiation

**Fig. 1:**
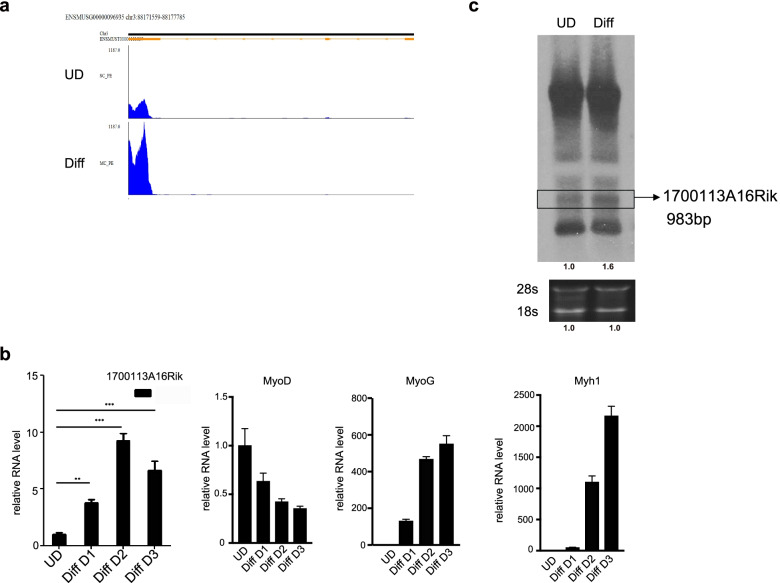
1700113A16Rik expression increased in differentiated muscle cells. **A** Deep-sequencing signals of 1700113A16Rik in the primary muscle stem cells (MuSCs) before and after differentiation. 1700113A16Rik located on mouse chromosome 3. (UD, undifferentiation; Diff, differentiation). **B** The relative RNA level of 1700113A16Rik, MyoD, myogenin, Myh1 in primary MuSCs and differentiated myotubes from day1 to day3 respectively. RT-qPCR assays were performed with RNA extracted from primary MuSCs and MuSCs differentiated from day1 to day3. The results were normalized to GPADH. Error bars indicated standard deviation and were based on 4 independent experiments. ** indicated *p* < 0.01, ***indicated *p* < 0.001. **C** Northern blot of 1700113A16Rik in primary MuSCs before and 3 days after differentiation. 18S and 28S served as an internal control. The numbers below each panel indicated relative signal intensity

To systematically identify lncRNAs involved in myogenesis, we carried out RNA sequencing (RNA-seq) of primary MuSCs and differentiated myotubes (3 days after differentiation). Among the newly identified lncRNAs, we noticed that the expression of 1700113A16RIK was significantly increased during the differentiation process of MuSCs (Fig. 1a). 1700113A16RIK was located on chromosome 3 of the mouse genome with a total length of 983 nucleotides, including 3 exons: 199 nucleotides in the first exon, 85 nucleotides in the second exon, and 699 nucleotides in the third exon (Fig. 5a). We further confirmed the expression of 1700113A16RIK by RT-qPCR. Consistent with RNA-seq analysis, RT-qPCR results showed that the expression of RNA1700113A16RIK was strongly upregulated during the differentiation of MuSCs (Fig. 1b). We further analyzed the expression of 1700113A16RIK in the process of MuSC differentiation by Northern blot. The expression of 1700113A16RIK was upregulated during the differentiation of MuSCs (Fig. 1c). Taken together, these results revealed the expression of 1700113A16RIK increased during MuSC differentiation.

### 1700113A16RIK mainly located in the cytoplasm of muscle cells

**Fig. 2:**
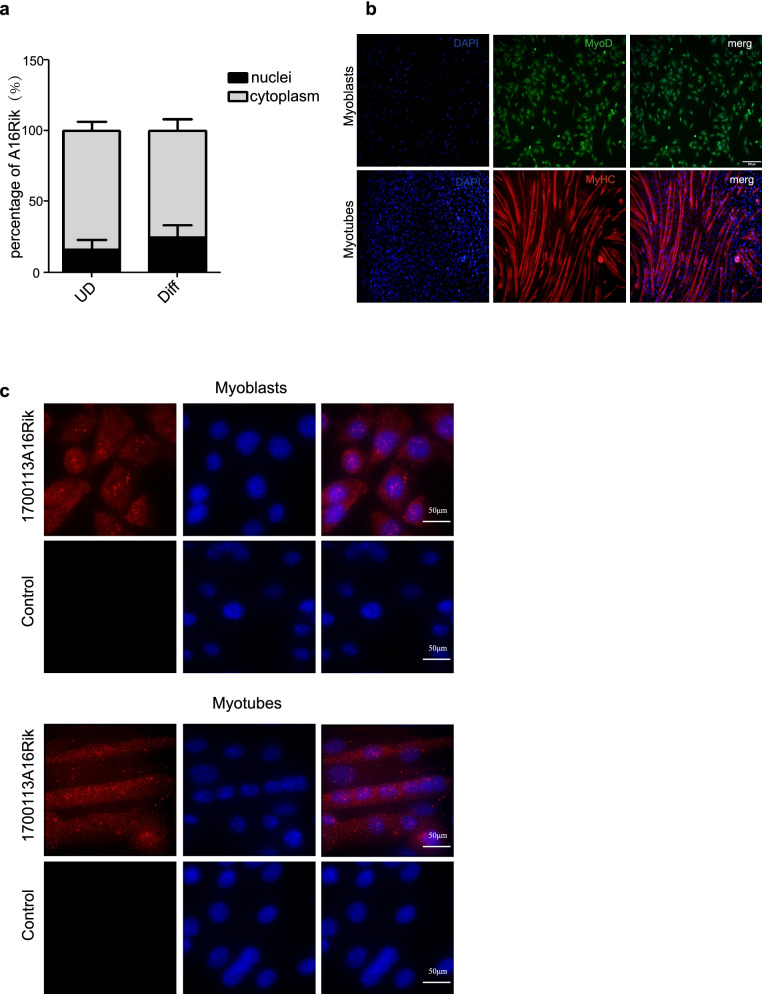
1700113A16Rik was localized in both cytoplasm and nucleus of muscle cells and enriched in the cytoplasm of muscle cells. **A** RT-qPCR was performed with RNA extracted from the cytoplasm and nucleus of MuSCs before and after differentiation to detect the distribution of 1700113A16Rik. 1700113A16Rik was upregulated during MuSC differentiation and enriched in the cytoplasm of MuSCs. Error bars indicated standard deviation and were based on 3 independent experiments. **B** Immunofluorescent staining of MyoD and MyHC. C2C12 myoblasts and the differentiated C2C12 cells were subjected for immunofluorescent staining with MyoD and MyHC antibody. Green indicated MyoD; Red indicated MyHC; Blue indicated DAPI; Merge indicated the merge of red, green and blue images. (Scale Bars, 200 μm). **C** RNA in situ hybridization was performed to detect the distribution of 1700113A16Rik in C2C12 cells before and after differentiation (Scale Bars, 50 μm)

We next examined the subcellular localization of 1700113A16RIK in MuSCs. The nuclear and cytoplasmic fractions were isolated from MuSCs and differentiated myotubes by nuclear/cytoplasmic RNA extraction kit. RNA was extracted from the nuclear and cytoplasmic fractions and subjected for RT-qPCR, and then detected the distribution of 1700113A16RIK expression. 1700113A16RIK transcript was mainly located in the cytoplasm of MuSCs and myotubes, though small amount of 1700113A16RIK RNA can also be detected in nuclei (Fig. 2a). To further verify the results, we performed RNA in situ hybridization (FISH) to detect the subcellular location of 1700113A16RIK in C2C12 cells. Immunofluorescence staining of MyoD and MyHC was used to mark cells before and after differentiation (Fig. 2b). Consistent with the RT-qPCR results from cell fractionation, the signal intensity of 1700113A16RIK in the cytoplasm of both myoblasts and myotubes was higher than that of in the nuclei (Fig. 2c). Collectively, these results suggest that 1700113A16RIK mainly located in the cytoplasm of muscle cells.

### Myogenin activates 1700113A16RIK after differentiation induction

**Fig. 3:**
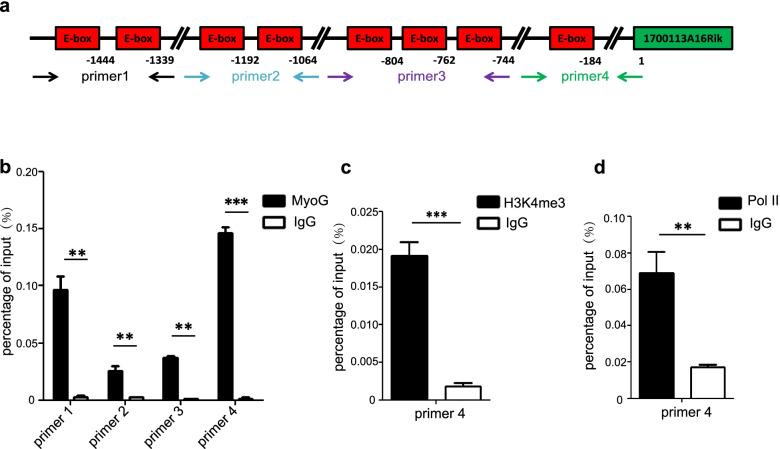
Myogenin positively regulated the expression of 1700113A16Rik. **A** Sequence analysis of E-box in the promoter region of 1700113A16Rik. **B** The binding efficiency of MyoG in the promoter region 1700113A16Rik. ChIP-qCPR was performed using MyoG antibody. Error bars indicated standard deviation and were based on 4 independent experiments. ** indicated p < 0.01, *** indicated *p* < 0.001. **C** The binding efficiency of H3K4me3 in the promoter region 1700113A16Rik. ChIP-qCPR was performed using H3K4me3 antibody. Error bars indicated standard deviation and were based on 4 independent experiments. *** indicated p < 0.001. **D** The binding efficiency of Pol II in the promoter region 1700113A16Rik. Error bars indicated standard deviation and were based on 4 independent experiments. ChIP-qCPR was performed using Pol II antibody. ** indicated *p* < 0.01

Previous results showed that 1700113A16RIK was upregulated after differentiation. We further investigated the transcription factor that transcriptionally regulated the expression of 1700113A16RIK in C2C12 cells. We analyzed the sequences of the promoter of 1700113A16RIK and found a cluster of eight E-box elements at the core promoter of 1700113A16RIK (Fig. 3a). E-box element can be recognized and bound by the key-differentiation transcription factor Myogenin (MyoG). We then performed MyoG, H3K4me3, and RNA polymerase II Chromatin immunoprecipitation (ChIP)-qPCR to detect their binding to the promoter of 1700113A16RIK. Four pairs of primers were designed to detect MyoG binding (Fig. 3a). The strongest MyoG binding was detected with primer 4, suggesting that the E box spanned by primer 4 has the highest affinity to MyoG (Fig. 3b). We therefore further tested the recruitment of H3K4me3 and RNA Pol II at the same region to confirm the transcription activation by MyoG. The enrichment of H3K4me3 and RNA Pol II at the promoter regions together with MyoG was detected, suggesting that MyoG activates 1700113A16RIK transcription after differentiation induction (Fig. 3c and d).

### 1700113A16RIK is required for muscle stem cells differentiation

**Fig. 4:**
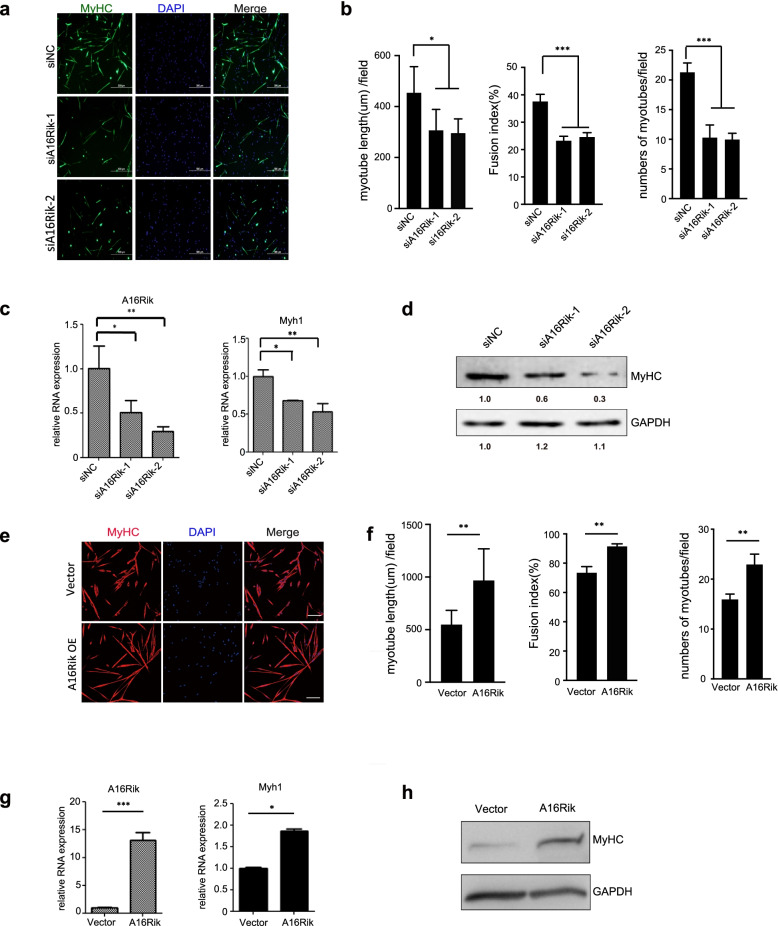
1700113A16Rik was required for the differentiation of MuSCs. **A** Immunofluorescent staining of MyHC in the control group (siNC) and 1700113A16Rik knockdown groups (si1700113A16Rik-1 and si1700113A16Rik-2). MuSCs were transfected by two pieces of siRNA against 1700113A16Rik or scramble RNA one day prior to differentiation and then differentiated for 3 days. The differentiated MuSCs were stained with anti-MyHC. Green indicated MyHC; DAPI indicated nuclear staining; merge indicated the merged images of green and blue. (Scale bars, 500 μm). **B** Statistical analysis of the number of MyHC positive cells, the myotube length and fusion index of myotubes described in A. Error bars indicated standard deviation and were based on 3 independent experiments. * indicated *p* < 0.05, *** indicated p < 0.001. **C** The relative RNA level of 1700113A16Rik and Myh1 in the differentiated MuSCs described in A. RT-qPCR assays were performed with RNA extracted from the differentiated MuSCs in the control group and the 1700113A16Rik knockdown group. Error bars indicated standard deviation and were based on 3 independent experiments. * indicated p < 0.05, ** indicated *p* < 0.01. **D** The protein level of MyHC in the differentiated MuSCs described in A. Western blot was performed from the differentiated MuSCs in the control group and the 1700113A16Rik knockdown group. The numbers below each panel indicated the relative signal intensity. **E** Immunofluorescent staining of MyHC. MuSCs were infected by control adenovirus and adenovirus encoding 1700113A16Rik and then differentiated for 3 days. The differentiated cells were subjected for immunofluorescent staining with MyHC antibody. Red indicated MyHC; Blue indicated DAPI; Merge indicated the merge of red and blue images. (Scale Bars, 200 μm). **F** Statistical analysis of the number of MyHC positive cells, the myotube length and fusion index of myotubes described in *E. Error* bars indicated standard deviation and were based on 3 independent experiments. * indicated p < 0.05, ** indicated *p* < 0.01

To investigate the function of 1700113A16RIK in myogenesis, we constructed two different pieces of siRNAs to target 1700113A16RIK. The expression of 1700113A16RIK was down-regulated significantly by the siRNAs (Fig. 4c). Knockdown of 1700113A16RIK led to differentiation defects in MuSCs as indicated by the decreased frequency of myotubes formation and shorter myotubes in differentiated MuSCs (Fig. 4a and b). Immunofluorescence staining showed that knockdown of 1700113A16RIK led to reduced number of MyHC positive cells after differentiation induction, and the fusion index also decreased (Fig. 4b). Consistent with the morphological changes, RT-qPCR results also showed that knockdown of 1700113A16RIK in MuSCs led to decreased expression levels of the late differentiation marker Myosin heavy chain 1 (Myh1) (Fig. 4c). Consistently, the protein levels of the late differentiation marker myosin heavy chain (MYHC) also decreased (Fig. 4d). Therefore, knockdown of 1700113A16RIK inhibited MuSC differentiation.

Then we constructed adenovirus encoding 1700113A16Rik to perform overexpression experiment in MuSCs (Fig. 4g). Overexpression of 1700113A16RIK promoted the differentiation of MuSCs as indicated by the increased frequency of myotube formation and longer myotubes (Fig. 4e and f). Immunofluorescence staining showed that overexpression of 1700113A16RIK increased the number of MyHC positive cells, the average myotube length and fusion index in differentiated MuSCs compared with the control group (Fig. 4f). RT-qPCR and Western blot results also showed that overexpression of 1700113A16RIK significantly upregulated the mRNA levels of Myh1 and the protein levels of MyHC during MuSC differentiation (Fig. 4g and h). These results suggested that enhanced expression of 1700113A16RIK promotes MuSC differentiation.

### 1700113A16RIK promotes muscle stem cells differentiation by facilitating Mef2d translation

**Fig. 5:**
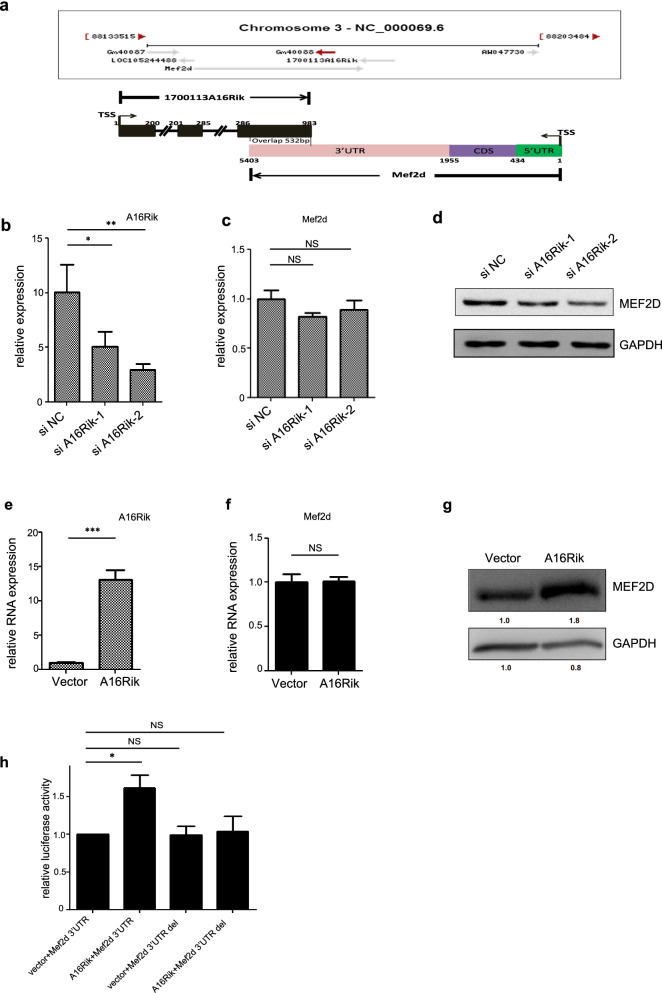
1700113A16Rik promoted MuSCs differentiation by targeting MEF2D. **A** The locations of 1700113A16Rik and MEF2D in the genome. The 3’UTR of Mef2d complementary paired with 1700113A16RIK. **B** The relative RNA level of 1700113A16Rik in the differentiated MuSCs. MuSCs were transfected by two pieces of siRNA against 1700113A16Rik (si1700113A16Rik-1 and si1700113A16Rik-2) or scramble RNA (siNC) and then differentiated for 3 days. RT-qPCR assays were performed with RNA extracted from the differentiated MuSCs in the control group and the 1700113A16Rik knockdown group. Error bars indicated standard deviation and were based on 3 independent experiments. * indicated *p*<0.05, ** indicated *p*<0.01. **C** The relative RNA level of Mef2d in the differentiated MuSCs described in **B**. RT-qPCR assays were performed with RNA extracted from the differentiated MuSCs in the control group and the 1700113A16Rik knockdown group. Error bars indicated standard deviation and were based on 3 independent experiments. NS indicated no significant changes. **D** The protein level of MEF2D in the differentiated MuSCs described in **B**. Western blot was performed from the differentiated MuSCs in the control group and the 1700113A16Rik knockdown group. **E** The relative RNA level of 1700113A16Rik in the differentiated MuSCs. MuSCs were infected by the control adenovirus and adenovirus encoding 1700113A16Rik and then differentiated for 3 days. RT-qPCR assays were performed with RNA extracted from the differentiated MuSCs in the control group and the 1700113A16Rikoverexpressing group. Error bars indicated standard deviation and were based on 3 independent experiments. *** indicated *p*<0.001. **F** The relative RNA level of Mef2d in the differentiated MuSCs described in **E**. RT-qPCR assays were performed with RNA extracted from the differentiated MuSCs in the control group and the 1700113A16Rik-overexpressing group. Error bars indicated standard deviation and were based on 3 independent experiments. NS indicated no significant changes. **G** The protein level of MEF2D in the differentiated MuSCs described in **E**. Western blot was performed from the differentiated MuSCs in the control group and the 1700113A16Rik-overexpressing group. The numbers below each panel indicated the relative signal intensity. **H** The luciferase activity of reporter gene carrying 1700113A16Rik target sequence from Mef2d at the 3’UTR region. Firefly luciferase activity regulated 1700113A16Rik target sequence at the 3’UTR. Renilla luciferase activity driven by CMV promoter served as an internal control. Error bars indicated standard deviation and were based on 5 independent experiments. * indicated *p*<0.05. NS indicated no significant changes

We next further explored the mechanisms of differentiation promoted by 1700113A16RIK. We carefully analyzed the sequence of 1700113A16RIK and found that a 532 nucleotides fragment in the third exon of 1700113A16RIK paired with the complementary sequence of the 3’UTR region of Mef2d (Fig. 5a). Mef2d is an important transcription factor in myogenesis which forms heterodimer with MRFs or directly binds to the promoter region of muscle-related genes to activate muscle cells differentiation (Sui et al. 2019). We next investigated the effect of 1700113A16RIK on the expression of Mef2d. Knockdown of 1700113A16RIK did not change the mRNA level of Mef2d as indicated by RT-qPCR assay in MuSCs (Fig. 5b and c). However, the protein level of Mef2d decreased significantly after knockdown of 1700113A16RIK (Fig. 5d). Similarly, overexpression of 1700113A16RIK in MuSCs did not affect the mRNA level of Mef2d while the protein level of Mef2d was significantly unregulated (Fig. 5e-g). Together, these results suggested 1700113A16RIK promotes the differentiation of MuSCs by facilitating the translation of Mef2d.

Then we explored how 1700113A16RIK promoted the translation of Mef2d. Sequence analysis revealed that the 3’UTR of Mef2d paired with 1700113A16RIK. The full length 3’UTR of Mef2d (Mef2d 3’UTR, about 3.5 kb) and the 3’UTR deletion with the 1700113A16RIK complementary sequence deleted (Mef2d 3’UTR del) were cloned into pGL3 luciferase vector. When the Mef2d 3’UTR luciferase reporter was co-transfected with 1700113A16RIK into MuSCs, the luciferase activity was increased (Fig. 5h). Deletion of the 1700113A16RIK complementary sequence abolished the effect of 1700113A16RIK (Fig. 5h). Together, these results identified 3’UTR of Mef2d as 1700113A16RIK target.

## Discussion

Myogenesis is tightly regulated in vivo by various transcription factors, including myogenic regulatory factors (MRFs) (MyoD, Myf5, MyoG and MRF4), the MEF2 family (Mef2a, −2c, and -2d), Pax3, and Pax7 (Lang et al. 2007; Potthoff and Olson 2007; Potthoff et al. 2007; Relaix et al. 2005; Rudnicki et al. 1993; Tajbakhsh et al. 1996; Zammit et al. 2006). In the recent decade, lncRNAs has been shown to play important roles in muscle differentiation as well. LncRNAs were involved in a variety of important cellular processes including chromatin remodeling, transcriptional regulation, post-transcriptional regulation, and as sponge for siRNAs to regulate multiple cellular processes (Watts et al. 2013; Borensztein et al. 2013; Dey et al. 2014; Wang et al. 2013; Legnini et al. 2014). However, the studies of lncRNAs are far less than those of proteins in myogenesis. To identify novel lncRNAs and study their functions will shed new lights on the regulation of myogenesis.

In our study, we found that lncRNA-1700113A16RIK mainly located in the cytoplasm of muscle cells. 1700113A16RIK was upregulated during muscle differentiation and promoted MuSC differentiation. More interestingly, we found that 1700113A16RIK conducted its differentiation promoting function by pairing to the 3’UTR of Mef2d to facilitate Mef2d translation.

This work provides a new paradigm of mechanism of how lncRNA regulating cellular processes. By pairing to the complementary sequence in Mef2d 3′ UTR, lncRNA may compete over microRNA targeting Mef2d and improve the translation. It may help recruit ribosomes to the 3’UTR of Mef2d to facilitate translation. To identify 1700113A16RIK interacting proteins will help pinpointing the mechanism of 1700113A16RIK mediated translational activation.

About 93% of the disease-related SNPs are located in the non-coding region (Hindorff et al. 2009). Compared with other coding RNAs, non-coding RNAs have a greater correlation with diseases related to genetic variation. For example, muscle-specific long non-coding RNA lnc-MD1 was associated with Duchenne muscular dystrophy (DMD). The expression of lnc-MD1 was extremely significantly reduced in the muscle cells of Duchenne muscular dystrophy mice (Cesana et al. 2011). Whether 1700113A16RIK plays an important role in muscular disease is also a question worthy of further study.

## Conclusions

We define 1700113A16RIK as a positive regulator of MuSC differentiation and elucidate a mechanism as to how 1700113A16RIK regulated MuSC differentiation.

## Methods

### Cell culture and differentiation

Primary MuSCs were isolated as previously described. Briefly, TA muscles from 3-months-old C57BL/6 mice were dissected and dissociated with collagenase (Roche, Indianapolis, IN, USA). The muscle cells in the flowthrough were subjected to CD34-FITC (BD biosciences) and integrin α_7_-allophycocyanin (R&D systems, Minneapolis, MN, USA) staining. The viable PI^−^CD34^+^integrin-α_7_^+^ MuSCs were collected by FACS sorting (Influx, BD biosciences, Franklin Lake, NJ, USA). MuSCs were cultured on collagen coated dishes in F10 medium containing 10% FBS, 5 ng/ml IL-1α, 5 ng/ml IL-13, 10 ng/ml IFN-γ, and 10 ng/ml TNF-α (R&D Systems), and 2.5 ng/ml FGF (Invitrogen, Red Wood City, CA, USA) as described previously. MuSCs were differentiated in differentiation medium (DMEM medium (invitrogen) containing 2% horse serum (HyClone, Malborough, MA, USA) for 3 days. C2C12 cells (ATCC) were cultured in DMEM medium (Invitrogen) containing 10% FBS (HyClone), and differentiated in DMEM medium (Invitrogen) containing 2% horse serum (HyClone) for 3 days. The differentiated myotubes were further isolated by pre-attaching to plates for 3 times.

### Cell transfection

Transient transfection of cells with siRNAs (Ribobio) was performed in 6-well plates using Lipofectamine® 3000 reagent (Life Technologies). Transient transfection of cells with DNA plasmids was performed in 6-well plates using Lipofectamine® LTX with Plus™ Reagent (Life Technologies). For genes functional analyses, all plasmids (4μg per well of 6-well plate) were transfected into cells in culture medium and then harvested for further detection.

### Immunofluorescent staining

Cells were fixed in 4% paraformaldehyde for 1 h and permeabilized in 0.25% Triton X-100 for 15 min at room temperature. The cells were blocked in 1% BSA for 30 min at room temperature and then incubated with anti-MyHC (Upstate, 05-716) and anti-MyoD (Santa Cruz, 5.8A) at 4 °C overnight with gentle shaking, followed by staining with Alexa 561-labeled anti-mouse antibodies (Invitrogen) at room temperature for 1 h, with three washes after each antibody incubation. Nuclei were counter-labeled with DAPI. All images were acquired by confocal microscopy (Leica, Wetzlar, Germany).

### Western blot

Cells were lysed in lysis buffer (50 mM Tris-HCl pH 7.4, 100 mM NaCl, 0.5% Tween-20, 0.5% NP-40 and protease inhibitors) on ice for 30 min. The supernatant was centrifuged for 30 min at 13500 rpm and 4 °C, ran on SDS–PAGE and then transferred to nitrocellulose membrane. The membranes were blocked with 5% non-fat milk for 1 h at room temperature and then incubated with anti-MyHC (Upstate, 05-716), anti-GAPDH (Cell signaling technology, 2118) antibodies, mouse anti-Mef2d (BD, 610774) at 4 °C overnight. Finally, secondary horseradish peroxidase-labeled antibody was added and incubated for 1 h at room temperature. Signals were detected with an enhanced chemiluminescence system (Thermo Scientific) and visualized by image analyzer.

### Real-time PCR analysis

Total RNA from tissues or cells was extracted in Trizol Reagent (Life Technologies) according to the manufacturer’s instructions. RNA (1μg) was treated with DNase I (NEB) and then reverse-transcribed to cDNA using the M-MuLV reverse transcriptase (NEB) according to the manufacturer’s instructions. Briefly, RNA was firstly denatured at 75 °C for 5 min. M-MuLV reverse transcriptase was then added and incubated at 42 °C for 1 h, and heated at 95 °C for 5 min. Quantitative PCR (qPCR) reactions were performed in triplicates using SYBR Green PCR master mix (DBI, Hazleton, PA, USA) in BioRad thermocycler system (BioRad, Herculase, CA) and analyzed by iQ5 optical system software (BioRad).

The primers for RT-qPCR and siRNA sequences are listed below:

- Mouse Myh1 F: TCGATGACCTCGCTAGTAACA
- Mouse Myh1 R: TTTCGTCTAGCTGGCGTGAG
- Mouse MEF2D F: CCGTTTCTCTCAGCAACCTC
- Mouse MEF2D R: CGGTCTCATAGGATCCTCCA
- Mouse GAPDH F: ACCCAGAAGACTGTGGATGG
- Mouse GAPDH R: ACACATTGGGGGTAGGAACA
- Mouse 1700113A6Rik F: GAGTAGTCCTGGGCCGCTAT
- Mouse 1700113A6Rik R: AGAGATGGCGGTGAAACTCG
- A16RIK siRNA-1:
- Sense: AUACGAGGCUAAGGGAAUU
- Anti-sense: AAUUCCCUUAGCCUCGUAU
- A16RIK siRNA-2:
- Sense: GCCAUCUCUUACUAGGAUU
- Anti-sense: AAUCCUAGUAAGAGAUGGC

### Nuclear and cytoplasmic RNA fractionation

Nuclear and cytoplasmic RNA fractionation was performed in MuSCs using a nuclear/cytoplasmic RNA extraction kit (Chunhe, Yangling, China). The detailed separation method is described in the manufacturer’s instructions. Nuclear and cytoplasmic RNA was extracted and transcribed into cDNA for further analysis.

### Dual-luciferase reporter assay

WT or Mut Mef2d 3’UTR were inserted into pmirGLO (Promega) at the 3′ end of the coding sequence of Firefly luciferase and then transfected into muscle stem cells. The activity of Firefly and Renilla luciferase was measured 48 h after transfection. Cells were harvested and lysed with lysis buffer. Firefly and Renilla luciferase activities were evaluated using the Luciferase assay systems (Promega, E1910) according to the manufacturer’s instructions. Firefly luciferase activity was normalized to the Renilla luciferase activity.

### Statistical analysis

Two-tailed Student’s test was performed for pairwise comparison between two groups. For multiple comparisons, a one-way ANOVA test was used, followed by Dunnett’s post-test when comparing each group to the control group, or followed by Tukey’s post-test when comparing all pairs of groups. Statistical analysis was performed in GraphPad Prism 7 (GraphPad Software) or Microsoft Excel (Microsoft). Differences are statistically significant at *P* < 0.05. *P < 0.05; **P < 0.01; ***P < 0.001; ns, no significance.

## Data Availability

All data supporting the findings of this study are available within the article. Materials request should be addressed to the corresponding author.

## Abbreviations

ChIP: chromatin immunoprecipitation
DMD: Duchenne muscular dystrophy
FISH: RNA in situ hybridization
LncRNA: long non-coding RNA
MEF2D: myocyte-specific enhancer binding factor 2
MRFs: myogenic regulatory factors
MuSCs: muscle stem cells
MyHC: myosin heavy chain
MyoG: myogenin
RT-qPCR: quantitative real-time PCR
